# Primary and Secondary/ Metastatic Salivary Duct Carcinoma Presenting within the Sinonasal Tract

**DOI:** 10.1007/s12105-020-01271-8

**Published:** 2021-01-11

**Authors:** Abbas Agaimy, Sarina K. Mueller, Justin A. Bishop, Simion I. Chiosea

**Affiliations:** 1 Institute of Pathology, Friedrich-Alexander-University Erlangen-Nürnberg, University Hospital Erlangen, Erlangen, Germany; 2 Department of Otorhinolaryngology, Head and Neck Surgery, Friedrich-Alexander-University Erlangen-Nürnberg, University Hospital Erlangen, Erlangen, Germany; 3 Department of Pathology, University of Texas Southwestern Medical Center, Dallas, TX USA; 4 Department of Pathology, University of Pittsburgh Medical Center, Presbyterian Hospital, Pittsburgh, PA 15213 USA

**Keywords:** Salivary duct carcinoma, Ductal adenocarcinoma, Non-intestinal adenocarcinoma, Carcinoma ex pleomorphic adenoma, Sinonasal tract, HER2/neu

## Abstract

Traditionally, sinonasal adenocarcinomas have been subdivided into intestinal (ITAC) and non-intestinal (non-ITAC) categories. The latter encompasses salivary-type adenocarcinomas originating from the seromucinous glands of the sinonasal mucosa and non-salivary adenocarcinomas. The non-salivary adenocarcinoma category is further subdivided into low-and high-grade variants. Among salivary-type sinonasal adenocarcinomas, tumors recapitulating salivary duct carcinoma (SDC) are exceedingly rare, but some might have been lumped into the high-grade non-ITAC category. To date, only three primary SDCs originating in the sinonasal tract have been reported. We herein describe 7 cases of SDC including one previously reported case (4 primary sinonasal, 3 metastatic/ extension from parotid gland SDC). The primary tumors affected 3 males and one female aged 60 – 75. Different sites were involved by the primary tumors while the secondary tumors affected the sphenoidal (2) and the frontal + maxillary (1) sinuses. Three primary tumors were de novo high-grade SDC and one was confined to contours of a pre-existing pleomorphic adenoma. All 3 secondary tumors were SDC ex pleomorphic adenoma of the parotid with a long history of recurrences, ultimately involving the sinonasal tract. Androgen receptor was positive in 7/7 cases. Four of 6 cases were strongly HER2/neu + (either score 3 + or with verified amplification). This small case series adds to the delineation of primary sinonasal SDC highlighting that almost half of invasive SDC presenting within sinonasal tract indeed represents extension or metastasis from a parotid gland primary. There is a tendency towards overrepresentation of HER2/neu-positive cases in both categories (primary and metastatic), but this needs clarification in larger studies.

## Introduction

As a consequence of extensive molecular studies utilizing innovate next generation sequencing tools, the classification of sinonasal carcinomas has received significant attention in recent years [1, 2]. Although most of recent studies were devoted mainly to sinonasal undifferentiated carcinoma (SNUC) and related poorly differentiated carcinomas, adenocarcinomas have received some attention as well.

Traditionally, primary sinonasal adenocarcinomas have been subdivided into two major categories: intestinal type (ITAC), related to occupational wood dust exposure, and non-intestinal (non-ITAC) adenocarcinomas [3, 4]. The non-ITAC category encompasses salivary-type adenocarcinomas originating from seromucinous glands of the sinonasal mucosa and non-salivary adenocarcinomas. The latter are further subdivided into low-and high-grade variants [3, 4].

With more studies, it became evident that the non-ITAC category is heterogeneous and reproducible diagnostic criteria have not been established yet [5]. Among salivary-type sinonasal carcinomas, adenoid cystic carcinoma significantly outnumbered other rare types while pleomorphic adenoma is the main benign tumor encountered [6]. To our knowledge, only three cases of genuine salivary duct carcinoma (SDC)-type sinonasal adenocarcinoma have been reported before, one by our group [7–9]. We herein present clinicopathologic and molecular features of 7 SDC-type adenocarcinomas presenting in the sinonasal tract and diagnosed via transnasal biopsies. Tumors were included irrespective of being primary or secondary, de novo or ex pleomorphic adenoma.

## Material and Methods

Cases were identified in the routine and consultation files of the authors. One case (Case 2 in Table 1) has been previously reported [8], but follow-up has been updated and molecular testing performed. The tumor specimens were fixed in buffered formalin and embedded for routine histological examination. Immunohistochemistry (IHC) was performed on 3-µm sections cut from paraffin blocks using a fully automated system (“Benchmark XT System”, Ventana Medical Systems Inc, 1910 Innovation Park Drive, Tucson, Arizona, USA) and the following antibodies: CK7 (OV-TL, 1:1000, Biogenex), CK5 (clone XM26, 1: 50, Zytomed), S100 protein (polyclonal, 1:2500, Dako), SOX10 (polyclonal, 1:25, DCS), androgen receptor (clone AR441, 1:50, DAKO), HER2/neu (polyclonal, 1:1000, DAKO), and SMARCB1 (INI1) (MRQ-27, 1:50, Zytomed). HER2/neu expression status was assessed using methods established for breast cancer and is considered positive when the Dako Score is 3 + . Cases scored 2 + (equivocal) were then subjected to chromogenic in situ hybridization (CISH) using a Zyto*Light* SPEC ERBB2/CEN 17 Dual Color Probe designed for the detection of *HER2/neu* amplification (ZytoVision, Bremerhaven, Germany) following recommendations of the manufacturer. Samples were used in accordance with ethical guidelines for the use of retrospective tissue samples provided by the local ethics committee of the Friedrich-Alexander University Erlangen-Nuremberg (ethics committee statements 24.01.2005 and 18.01.2012).

**Table 1:** Clinicopathological features of current and reported salivary duct carcinoma presenting within sinonasal tract (n = 9)

No	Case Ref.	Age/ Gender	Involved sinonasal sites	Histology	Site of primary tumor	Other tumors	TNM primary sinonasal SDC	Treatment	Outcome
1	Higo et al. [7]	73/M	Rt maxillary/ethmoid	SDC, high-grade, de novo	Sinonasal	No	NA	Paliative	Multiple lung mets, AWD (6 mo)
2	Current (Mueller et al.) [8]	60/M	Rt maxillary	SDC, high-grade, de novo	Sinonasal	No	pT2 pN2c (45/55) L1 V1 Pn0	Surgery + aCRT	Bone metastasis, DOD (35 mo)
3	Vallabh, et al. [9]	76/M	Rt inferior turbinate	SDC, high-grade, de novo	Sinonasal	No	NA	Surgery + aCT	ANED (18 mo)
4	Current	75/M	Nasal cavity (primary)	SDC, high-grade, de novo	Sinonasal	No	T2NxMx	Surgery	NA
5	Current	71/M	Rt sphenoid sinus	SDC ex PA (confined to the PA)	Sinonasal	Concurrent inverted sinonasal papilloma, maxillary sinus	TisNxMx	Surgery	ANED (24 mo)
6	Current	62/F	Maxillary sinus/ nasal floor	SDC, high-grade, de novo	Sinonasal	NA	pT4 N2b (AJCC 7) or N3b (AJCC 8)	Surgery + CRT	NA
7	Current	57/M	Left sphenoidal sinus	SDC ex PA, high-grade	Parotid	SDC ex PA parotid	–	CRT	1994: parotid gland PA 1998 and 2005: PA recurrences 2020: SDC ex PA with sinonasal involvement
8	Current	56/M	Left sphenoidal sinus	SDC ex PA, high-grade	Parotid	SDC ex PA parotid	–	Surgery and CRT	SDC ex PA parotid (surgery + CRT) Bone mets (9 mo later) Pterygopalatine fossa spread and sinonasal involvement (24 mo later)
9	Current	64 /F	Frontal and maxillary sinuses	SDC ex PA, high-grade	Parotid	SDC ex PA parotid	–	Surgery and CRT	2009: parotid SDC ex PA (surgery + CRT) 2014: recurrence in pre-molar/buccal space 2018: sinonasal involvement and distant mets to lung

*Clinical data incomplete to reliably rule out a salivary gland primary

*aCRT* adjuvant chemoradiotherapy, *aCT* adjuvant chemotherapy, *ANED* alive with no evidence of disease, *AWD* alive with disease, *CRT* chemoradiotherapy, *CT* chemotherapy, *DOD* died of disease, *F* female, *M* male, *mets* metastasis, *NA* not available, *PA* pleomorphic adenoma, *SDC* salivary duct carcinoma, *Rt* right

### Molecular Testing

#### DNA Testing

Different molecular next generation sequencing (NGS) panels targeting DNA sequence variants (mutations) were used on different cases [for Cases 6 and 9 see ref. 10].

To analyze the mutational status of common cancer related genes, DNA was isolated from FFPE tissue sections (Case 8) using the Maxwell 16LEV Blood DNA kit (Promega, Madison, USA) and submitted to hybrid-capture enrichment-based sequencing analysis using the TruSight Tumor 170 (TST170) gene panel (Illumina, Inc., San Diego, CA, USA) according to the manufacturer`s protocol. Libraries were sequenced on a Next Seq550 (Illumina) and analyzed for single nucleotide mutations, insertions, deletions and copy number variations using the TruSight Tumor 170 software (BaseSpace Sequence Hub, Illumina) with human genome hg19 as reference.

#### RNA Testing

For Cases 2 & 6—9, RNA was isolated from formalin-fixed paraffin embedded (FFPE) tissue sections using RNeasy FFPE Kit of Qiagen (Hilden, Germany) and quantified spectrophotometrically using NanoDrop-1000 (Waltham, United States). Molecular analysis for gene fusions was performed using the TruSight RNA Fusion panel (Illumina, Inc., San Diego, CA, USA) with 500 ng RNA as input according to the manufacturer`s protocol. Libraries were sequenced on a MiSeq (Illumina, Inc., San Diego, CA, USA) with > 3 million reads per case, and sequences were analyzed using the RNA-Seq Alignment workflow, version 2.0.1 (Illumina, Inc., San Diego, CA, USA). The Integrative Genomics Viewer (IGV), version 2.2.13 (Broad Institute, REF) was used for data visualization. Case 5 was tested for gene fusions and sequence variants using the method described recently [11].

## Results

Of 8 cases identified initially for this study (Cases 2 and 4–9 in Table 1), one *HER2/neu*-amplified, androgen receptor-negative high-grade adenocarcinoma was excluded due to lacking detailed clinical data and imaging to rule out a salivary gland tumor or other primary. This tumor showed extensive colonization of adjacent seromucinous glands and ducts suggesting a primary sinonasal origin. Four of the remaining 7 tumors (57%) were primary sinonasal and 3 represented discontinuous metastasis or contiguous extension from SDCs of the parotid gland.

### Primary Sinonasal Salivary Duct Carcinomas

The 4 patients with definite primary sinonasal SDC were 3 males and one female aged 60 – 75 years. Site of origin was maxillary sinus (2), sphenoid sinus (1) and nasal cavity (1). None had evidence of another primary tumor in the head and neck or other organs at the time of diagnosis. Three tumors were invasive high-grade de novo SDC and one was a high-grade SDC confined to the contours of the preexisting pleomorphic adenoma. This latter patient had a concurrent inverted sinonasal papilloma in the maxillary sinus not related to the site of his SDC ex pleomorphic adenoma. The invasive tumors were T2-4; two of them had extensive synchronous cervical node metastases (N2b-N3). Treatment was surgery in all cases; two received adjuvant chemoradiation. Follow-up was available for two patients. One patient developed bone metastases; he died of his disease 35 months from initial diagnosis. The patient with SDC ex pleomorphic adenoma remained disease-free 24 months later.

### Pathological and Immunohistochemical Findings

**Fig. 1:**
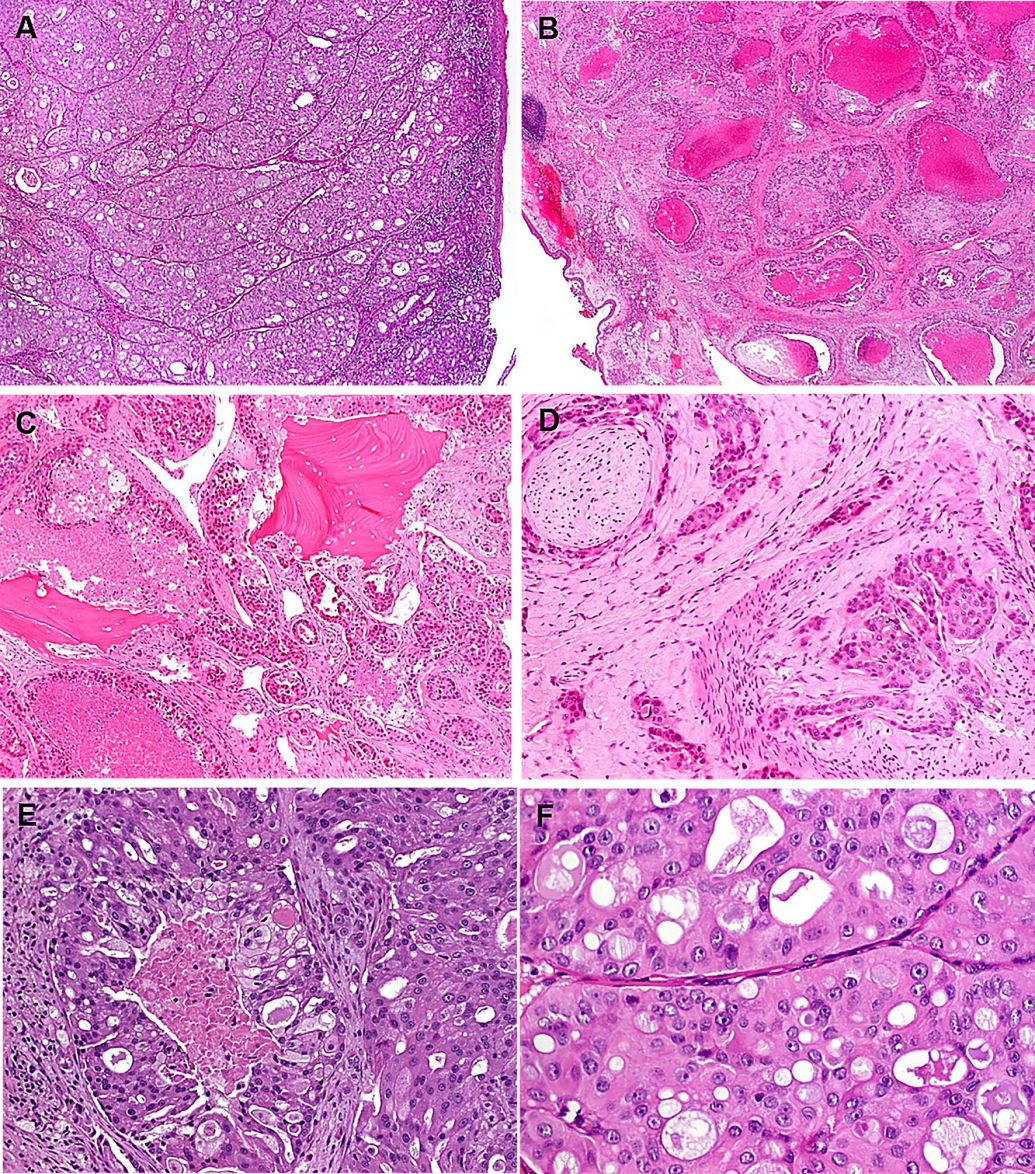
Representative images of primary sinonasal salivary duct carcinomas. At low power, SDC infiltrates and replaces the lamina propria with retained respiratory epithelial covering at the surface with variable reactive squamous metaplasia. Note variation from diffuse solid and sieve-like growth **a** to well defined large DCIS-like nests with extensive comedo-type necrosis. **b** Destructive invasion of underlying bone **c** and perineural and angioinvasion **d** are seen. **e** transition from classical SDC pattern (left) to solid/adenoid pattern (right). **f** high-grade apocrine cytology is appreciated at high power

**Fig. 2:**
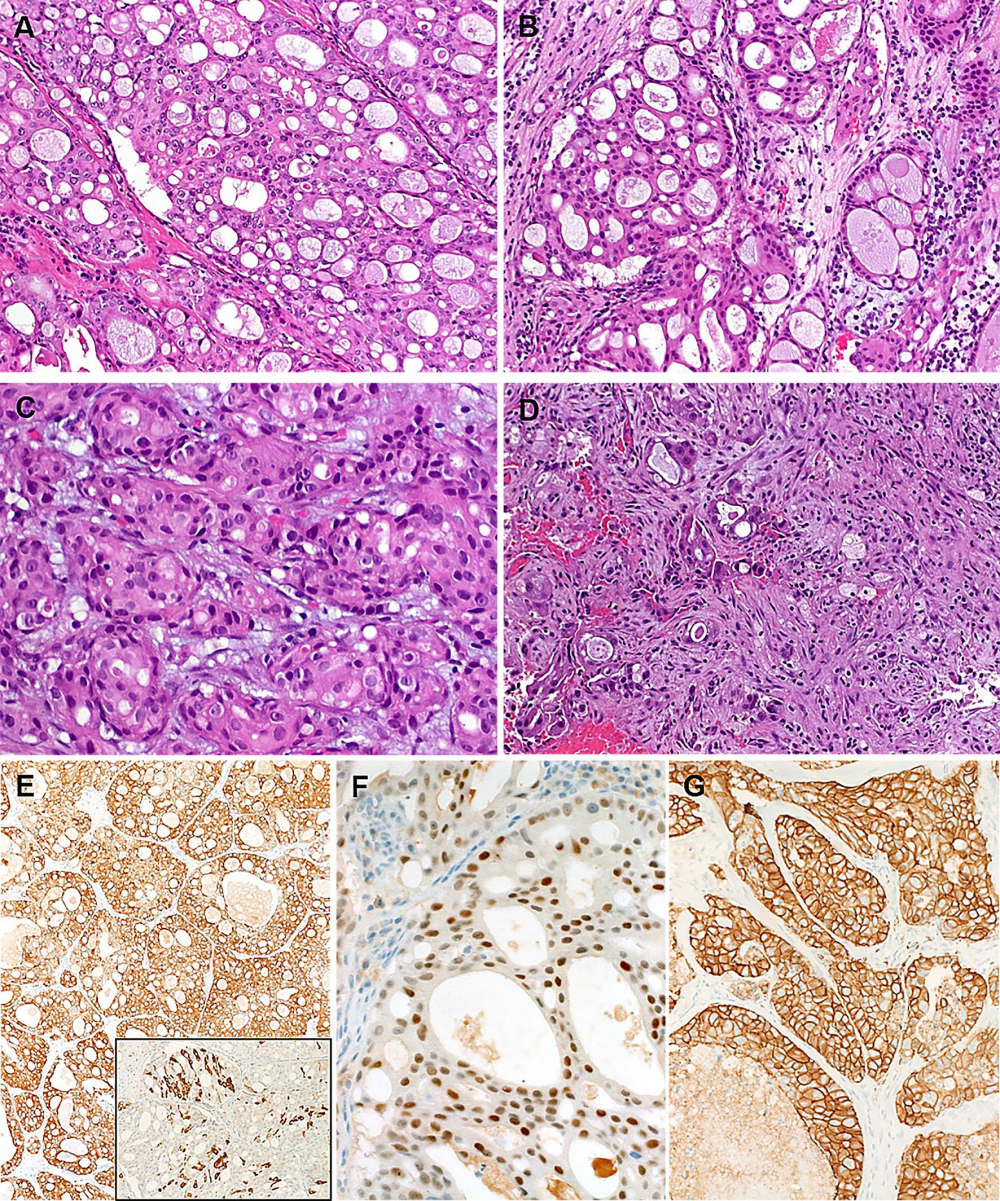
Variant SDC patterns were seen to variable extent in most of cases including diffuse solid-sieve-like mimicking secretory carcinoma **a**, **b**, small nested pattern **c** and less differentiated poorly cohesive small nests and single cells amid desmoplastic stroma **d**. By immunohistochemistry, all cases expressed diffusely CK7 (**e**, main image), androgen receptor (**f**) and HER2/neu (**g**). Mammaglobin was positive in scattered cells or cluster of cells (E, subimage)

**Fig. 3:**
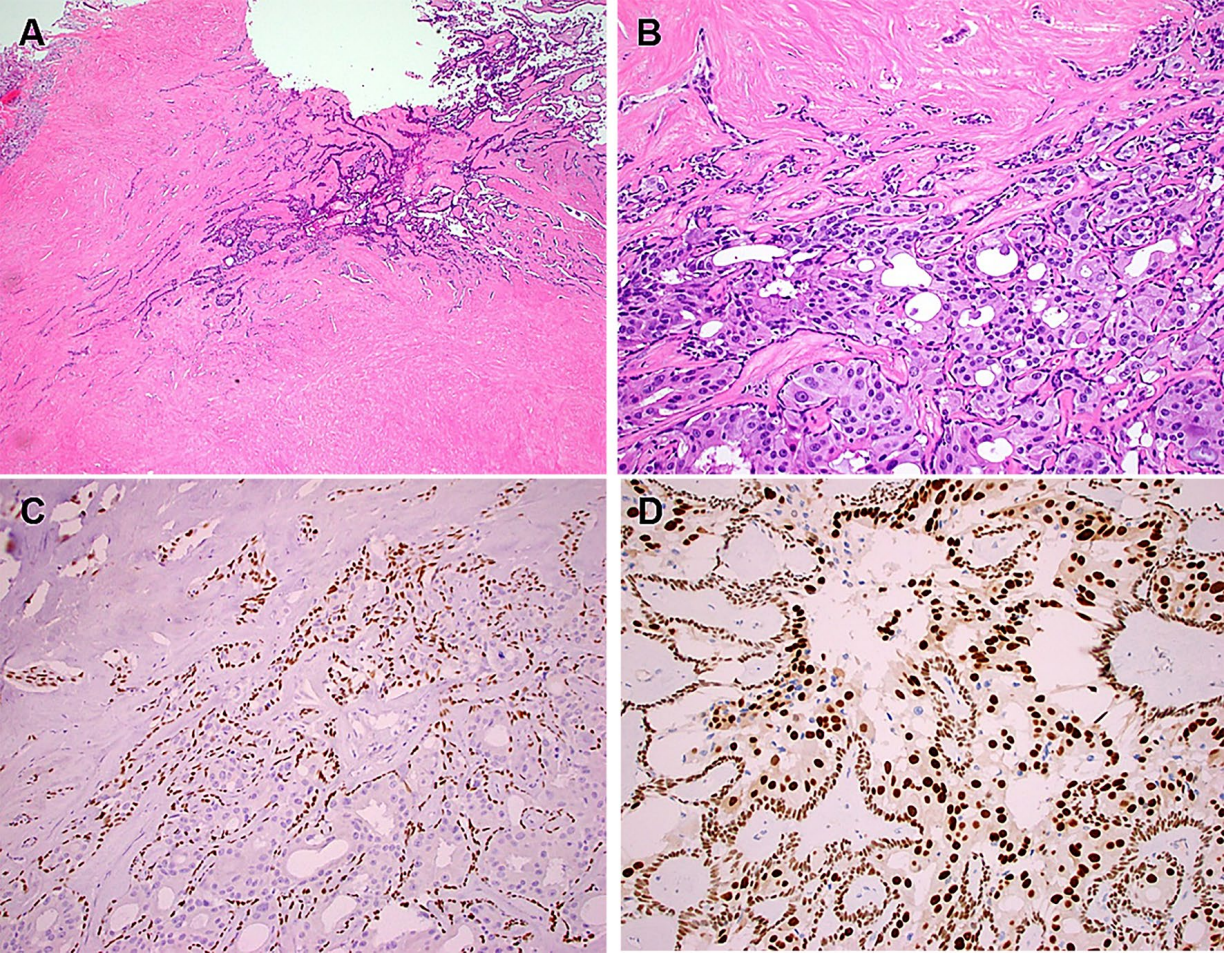
The single case of SDC ex pleomorphic adenoma showed areas of classical pleomorphic adenoma blending with extensive sclerosis **a** and confluent areas of highly atypical apocrine-type cells confined to the contours of the preexisting adenoma **b** and surrounded by intact layer of smooth muscle actin + /p40 + basal/myoepithelial cells (**c**; p40 immunostain). **d** the carcinoma cells are strongly positive with the androgen receptor

Histologically, all primary invasive SDC showed classical histology of the entity with predominance of ductal, cribriform-like and irregular nests invading through the sinonasal mucosa with high-grade apocrine cytology and prominent foci of comedonecrosis (Fig. 1a-f). In addition to the classical SDC pattern, foci of variant histology were seen in all three cases, but to variable extent, including sieve-like solid growth in two cases (Fig. 2a, b), small nested pattern in one (Fig. 2c) and minor pleomorphic poorly cohesive foci in all cases (Fig. 2d). All tumors, expressed diffusely and strongly CK7 (Fig. 2e main image) and the androgen receptor (Fig. 2f), but lacked expression of CK5, S100 and SOX10 (Table 2). Mammaglobin was positive in a few single cells or group of cells in 3 of 3 cases (Fig. 2e, inset). Two of 3 tumors tested for HER2/neu showed strong membranous expression indicating amplification (score 3 + ; Fig. 2g). The third tumor (scored 2 +) revealed no amplification by CISH. All 4 tumors showed retained expression of SMARCB1/INI1. An intraductal/intraepithelial component was seen only in the one SDC ex pleomorphic adenoma and was so prominent, suggesting an initial diagnosis of oncocytic sinonasal papilloma by the submitting pathologist (Fig. 3).

**Table 2:** Immunohistochemical and molecular features of current and reported salivary duct carcinoma presenting within sinonasal tract (n = 9)

No	Case Ref	CK7	CK5	AR	HER2neu	S100	SOX10	RNA Panel	DNA testing
1	Higo et al. [7]	ND	+	+	ND	ND	ND	ND	ND
2	Current (Mueller et al.) [8]	+	−	+	2 + (CISH-)	−	−	Failed (poor RNA quality)	ND
3	Vallabh, et al. [9]	+	Focal	ND	ND	ND	ND	ND	ND
4	Current	+	−	+	3 +	−	−	ND	ND
5	Current	+	−	+	ND	−	−	No fusions but HRAS p.Q61K	ND
6	Current	+	−	+	3 +	−	−	Failed (poor RNA quality)	Wild type PIK3CA
7	Current	+	−	+	3 +	−	−	Failed (poor RNA quality)	ND
8	Current	+	−	+	3 + (CISH +)	−	−	HFM1/ETV1 fusion	TST170: p53 mutation (p.Gly245Ser)
9	Current	+	−	+	−	−	−	Failed (poor RNA quality)	PLAG1 & HMGA2 intact by FISH, 150 gene panel: PIK3CA E542K and HRAS Q61K mutations;

*ND* not done

### Molecular Results

RNA fusion panel was tried in 3 primary tumors with sufficient material; but it failed in two cases due to poor RNA quality (Table 2). The case of primary SDC ex pleomorphic adenoma revealed no fusion but sequence assessment showed a *HRAS* mutation (p.Q61K).

### Secondary Sinonasal Salivary Duct Carcinomas

**Fig. 4:**
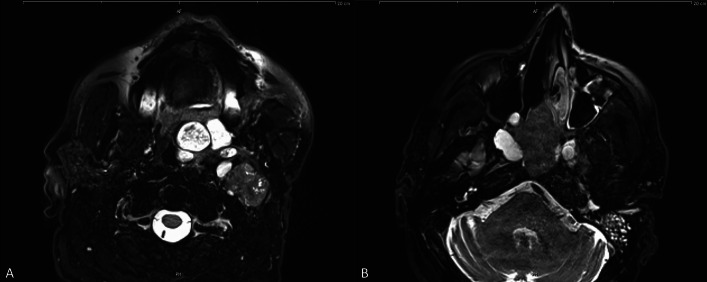
Representative imaging of SDC ex pleomorphic adenoma with secondary involvement of the sinonasal tract (Case 7). Axial T2-weighted MRI scans showing a heterogeneous tumor in the left parotid region **a** extending into the nasopharynx and into the sinonasal tract. **b** The nodules with high signal intensity correspond to the recurrent pleomorphic adenoma, while the mass with attenuated signal intensity extending into the sinonasal cavities represents the SDC component (verified by histology)

The three patients with parotid gland SDC involving the sinonasal tract were 2 males and one female aged 56, 57 and 64 years (Cases 7–9 in Table 1). All three had high-grade SDC ex pleomorphic adenoma. They all experienced at least one locoregional recurrence before presentation with sinonasal manifestation. One patient with a history of parotid gland pleomorphic adenoma for more than 20 years had resection of recurrent pleomorphic adenoma 15 years ago. He then presented with extensive disease with involvement of the parapharyngeal space and the left sphenoidal sinuses (Fig. 4). The diagnosis of SDC ex pleomorphic adenoma was rendered on sinonasal biopsies. One patient had distant spread to the lung.

### Pathological and Immunohistochemical Findings

**Fig. 5:**
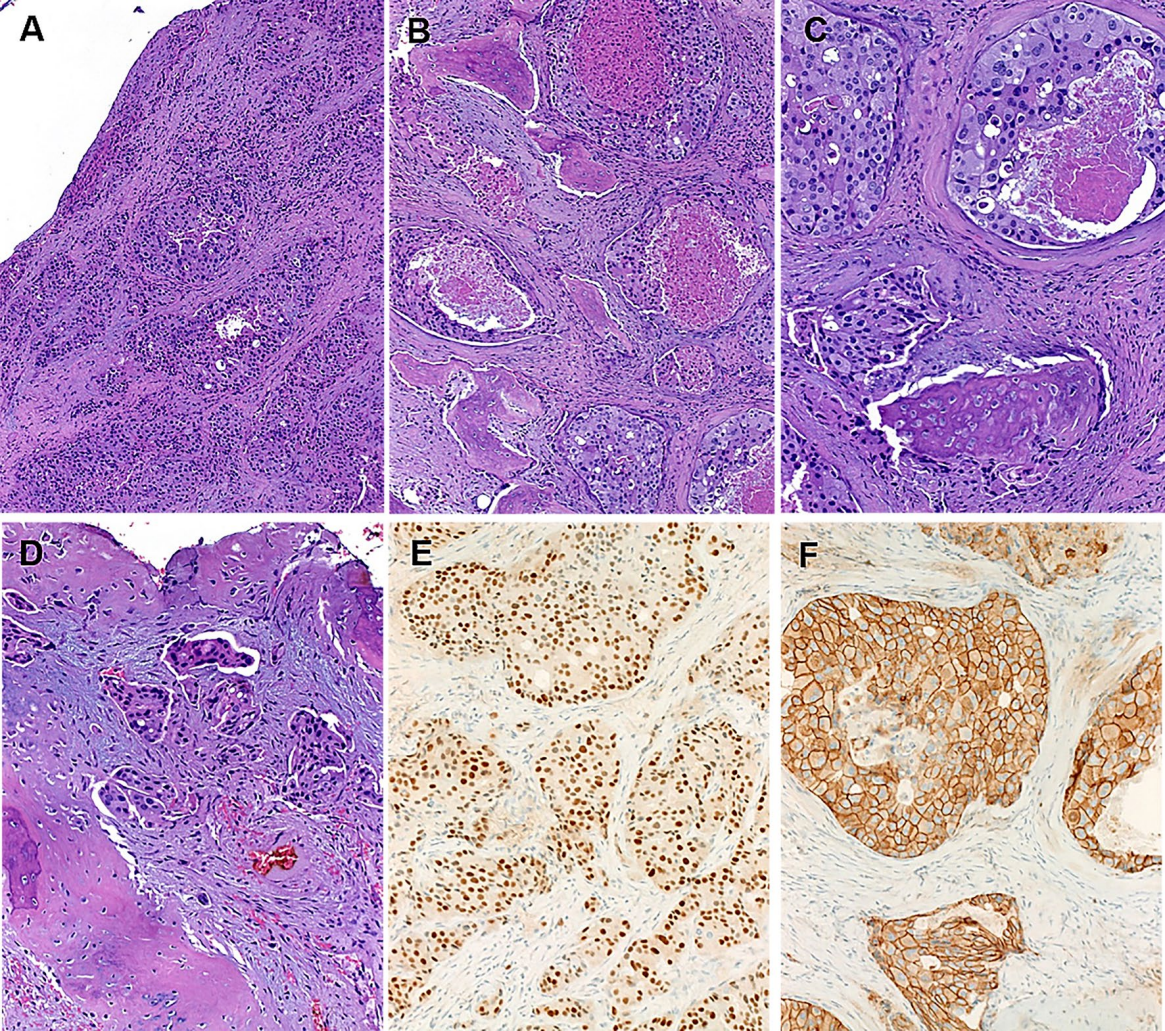
Secondary/ metastatic SDCs within the sinonasal sinuses are essentially indistinguishable from primary tumors with diffuse infiltration beneath eroded surface epithelium **a**, extensive bone invasion **b** and high-grade apocrine cell morphology with prominent comedonecrosis. **c** Micropapillary-like poorly differentiated foci are seen and might be misinterpreted as other-type high-grade carcinoma on biopsies. **d** All cases expressed the androgen receptor (**e**) and two of three cases HER2/neu (**f**)

Histologically, all secondary SDC showed very similar histology as their primary counterparts with predominance of ductal, cribriform-like and irregular nests invading through the sinonasal mucosa (Fig. 5a-d). Intraductal/intraepithelial growth was not seen in any case. All three tumors expressed diffusely and strongly CK7 and the androgen receptor (Fig. 5e), but lacked expression of CK5, S100 and SOX10. Two tumors showed strong HER2/neu overexpression (score 3 + ; Fig. 5f); CISH was performed on one of them (upon clinical request) and confirmed a high level *HER2/neu* amplification. All tumors showed retained expression of SMARCB1/INI1.

### Molecular Results

The three secondary tumors were tested for gene mutations and gene fusion by different DNA and RNA panels (see Table 2). The RNA panel failed in two cases due to poor RNA quality of the available paraffin material. One tumor showed an *HFM1-ETV1* fusion which has not been reported previously so that its oncogenic role remains unclear. DNA testing revealed typical gene mutations involving *p53* (one case) and *PIK3CA* + *HRAS* (one case). *PLAG1* and *HMGA2* status was assessed by FISH in one case of SDC ex pleomorphic adenoma and was negative for rearrangements.

## Discussion

Due to the lack of defining genetic markers, precise subtyping of sinonasal non-intestinal adenocarcinomas (non-ITAC) is still challenging. This significantly heterogeneous group encompasses bland looking low-grade (frequently tubulopapillary) adenocarcinomas and poorly characterized high-grade adenocarcinomas [5, 12, 13]. Recently, recurrent *ETV6-NTRK3* fusions have been described in a small subset of tumors in the spectrum of low-grade non-ITAC. Morphological characterization of the *ETV6*-fusion positive tumors revealed two different phenotypes: (1) tumors indistinguishable from (mammary-analogue) salivary gland secretory carcinomas which have been classified as primary sinonasal salivary-type secretory carcinomas [14, 15], thus adding to the list of salivary-type sinonasal carcinomas, and (2) tumors that belong to the spectrum of low-grade tubulopapillary adenocarcinoma and are distinct from secretory carcinomas, but harbor the same *ETV6* gene fusion [16]. The latter may harbor other rare gene fusions, though their molecular pathogenesis is still emerging [17].

With the recent characterization of the SMARCB1-deficient sinonasal carcinoma [18], a variant showing frankly glandular growth and occasional yolk sac-like pattern has been proposed as a distinctive variant in the spectrum of SWI/SNF-deficient sinonasal carcinomas [19]. This rare tumor has been likely included in the spectrum of high-grade non-ITAC in the past, although some might have been included in the oncocytic or myoepithelial carcinoma category due to their frequent “pink cytology” [19].

Salivary duct carcinoma (SDC; synonym: high-grade ductal carcinoma) is a highly aggressive malignancy of the excretory duct system accounting for up to 10% of salivary gland malignancies [20]. It mainly originates in the parotid gland and much less frequently from the submandibular gland [21]. Exceedingly rare reports from other sites include the minor salivary glands (mainly of the palate) [22], the Stensen duct [23], the lacrimal glands [24] and the larynx [25–27]. About 40–45% of SDC cases originates from primary or recurrent pleomorphic adenomas as carcinoma ex pleomorphic adenoma variant [10, 20, 21]. Defining features of SDC includes prominent ductal proliferation with comedo-type necrosis (high-grade DCIS-like growth pattern) admixed with other diverse patterns that recapitulate the different morphologies of high-grade invasive ductal breast cancer [28, 29]. Their cytology is frequently apocrine or oncocytoid [28, 29]. Expression of the androgen receptor is detected in 70% and HER2/neu is overexpression in 25–30% of cases, reflecting gene amplification [28–32].

SDC originating primarily in the sinonasal tract is rare but might be underecognized or misclassified in the generic spectrum of high-grade non-ITAC in the past. To date, only three genuine cases have been reported [7–9]. Among 115 sinonasal and nasopharyngeal pleomorphic adenomas and carcinomas ex pleomorphic adenoma identified in a recent literature review, 10 cases of carcinoma ex pleomorphic adenoma involved sinonasal sites; 5 of them were reported as “adenocarcinomas, NOS” [33]. There was no mention of SDC in that review study. These observations suggest that primary sinonasal SDC are likely more common than the reported cases suggest and some might have been either included in the high-grade non-ITAC group or misclassified as other entities.

Hence, the current series adds to the growing spectrum of salivary-type sinonasal adenocarcinoma. The demographic features and the clinicopathological characteristics of primary sinonasal SDC cases are comparable to those of salivary gland SDC. Moreover, metastatic and secondary SDC within the sinonasal tract are indistinguishable from primary tumors by morphology alone and the clinical history and imaging are mandatory for correct diagnosis. Study of additional cases is needed to shed light on possible overrepresentation of de novo origin (seen in 1 of 4 primary tumors) and of HER2/neu overexpression (observed in 2 of 3 primary tumors) in sinonasal SDC. The low number of cases in our study does not allow for conclusive results regarding these two points.

Our study highlights the frequency of sinonasal involvement by SDC originating primarily in the parotid gland with 3 of our 7 collected cases with complete clinical data being secondary SDC. We are not aware of similar documentation of SDC secondarily involving the sinonasal tract. Although larger series are needed for conclusive results, it is likely that SDC ex pleomorphic adenoma are more prone to sinonasal involvement (in the setting of multiple locoregional recurrences with ultimate extension or spread to the sinonasal tract) than de novo parotid gland SDC. In addition to metastasis from SDC of salivary gland origin, metastatic adenocarcinoma from different organs should always be considered in any sinonasal carcinoma not fitting the common types encountered as this site [34]. Given the predominance of older males, the major differential diagnostic consideration of primary and secondary sinonasal SDC is metastatic high-grade prostatic adenocarcinoma, as the two entities may share some morphological features and both express the androgen receptor [35]. In the appropriate clinicopathological context, inclusion of highly sensitive and specific prostate markers such as NKX3.1 is highly valuable compared to the traditional less sensitive prostate markers [36].

Extensive growth along preexisting glands (seen in the primary sinonasal SDC ex pleomorphic adenoma case) may closely mimic oncocytic sinonasal papilloma or carcinoma ex oncocytic sinonasal papilloma, underlining the necessity of careful assessment to rule out preexisting papilloma. Indeed, this case was initially submitted with a diagnosis of oncocytic sinonasal papilloma. The misleading pattern of carcinomatous growth along preexisting glands and ducts in the sinonasal mucosa was observed in other types of sinonasal carcinoma as well, frequently suggesting a “carcinoma ex sinonasal papilloma” [18].

In summary, we described a series of 4 primary and 3 secondary/metastatic sinonasal salivary duct carcinomas and reviewed previously reported single cases. Together, a total of 6 definite primary sinonasal SDC have been documented to date; all but one case originating de novo. Inclusion of salivary duct carcinoma in the differential of high-grade non-intestinal adenocarcinoma should facilitate recognition and hence better characterization of this rare highly aggressive malignancy.
